# Obesity and Kidney Transplant Candidates: An Outcome Analysis Based on Body Mass Index

**DOI:** 10.7759/cureus.34640

**Published:** 2023-02-05

**Authors:** Abdulrahman R al Tamimi, Rayan S Bahashwan, Saad A Almousa, Abdulaziz Aldalaan, Mohammed H Almusallam, Nawaf K Alawad, Abdullah F Alangari

**Affiliations:** 1 Organ Transplant Surgery, King Abdulaziz Medical City, King Abdullah International Medical Research Center Medical Research, King Saud Bin Abdulaziz University for Health Sciences, Riyadh, SAU; 2 Medicine, King Saud Bin Abdulaziz University for Health Sciences College of Medicine, Riyadh, SAU; 3 Medicine and Surgery, King Saud Bin Abdulaziz University for Health Sciences College of Medicine, Riyadh, SAU

**Keywords:** overweight and obesity, body mass index(bmi), end stage renal disease (esrd), diabetes mellitus, complications, kidney transplant, obesity

## Abstract

Background

Obesity is a well-established risk factor for a decline in renal function and post-operative complications. Also, obese patients suffer worse outcomes such as higher rates of wound complications, longer hospital stays, and delayed graft function (DGF) when compared to nonobese patients.

The correlation between having a high BMI and the postoperative outcomes of kidney transplantation has not been investigated yet in Saudi Arabia. There is scarce evidence that patients with obesity who have undergone kidney transplantation are devoid of any complications before, during, or after their procedure.

Methodology

A retrospective cross-sectional study was conducted using charts of nearly 142 patients in King Abdullah Specialist Children's Hospital in Riyadh, who had kidney transplant surgery in the organ transplantation department. All Obese patients with BMI >29.9 who underwent Kidney Transplant Surgery in King Abdulaziz Medical City from 2015 to 2022 were used. Details of hospital admissions were retrieved.

Results

A total of 142 patients fulfilling the inclusion criteria were included. There was a significant difference between patients regarding pre-surgical history where all cases (100%; 2) with class three obesity were hypertensive and on dialysis versus (77.8%; 21) and (70.4%; 19) of class two obesity and (86.7%; 98) and (78.8%; 89) of class one obesity cases, respectively (P = 0.041). Regarding medical history, hypertension was reported among 121 (85%), followed by dialysis (77%; 110), diabetes mellitus (DM) (52%; 74), dyslipidemia (24%; 35), endocrine diseases (15%; 22), and cardiovascular diseases (16%; 23).

Considering post-transplant complications, 14.1% (20) of the study cases had DM (16.8% of obese class one, 3.7% of obese class two, and none of obese class three; P = 0.996) and urinary tract infection (UTI) among 7% (10) of the cases (6.2% of obese class one, 11.1% of obese class two, and none of obese class three; P = 0.996). All these differences according to patients' BMI were statistically insignificant.

Conclusion

Obese patients are more likely to experience difficult intraoperative management along with a complicated postoperative course due to numerous concomitant comorbidities. Post-transplant DM (PTDM) was the most prominent post-transplant complication followed by UTI. A remarkable reduction in serum creatinine and blood urea nitrogen (BUN) has been observed at the time of discharge and after six months compared to pre-transplant measurements.

## Introduction

Obesity, the incidence of which has risen sharply over the past decade encompassing 24.7% of the Saudi population [1], is a well-established risk factor for worsening kidney function in both healthy individuals and those with a history of kidney transplantation [2]. Besides the current local prevalence, it is also estimated that by 2030, 38% of the world’s adult population will be overweight and 20% will be obese [3]. Obesity is widely assessed by the body mass index (BMI), defined as weight in kg/height in meters squared in adults, and the BMI percentile adjusted for age and sex in children, although it is argued that no standardized definition is applicable for the pediatric population. Despite the definition of obesity as excessive adiposity, there is no consensus on how to define obesity using fat mass calculation or fat percentage. The American Society of Clinical endocrinologists defines obesity as a body fat percentage of>35% in women and >25% in men [4].

Normative research has historically examined the wide-ranging complications and comorbidities of obesity in which kidney disease is rather remarkable. A study suggested that visceral obesity is an independent risk factor for end-stage renal disease (ESRD). It is also associated with a rise in relative risk with increasing body mass index (BMI) [5], as demonstrated by the increasing number of obese patients who have ESRD and are registered for kidney transplantation.

Next, post-operative complications and outcomes are worse in obese patients when compared with their non-obese counterparts, namely an increased risk of delayed graft function (DGF) with obesity [6]. However, even though morbidly obese patients have demonstrated some benefit from kidney transplantation compared to their medically treated counterparts [7], Weight bias in kidney transplantation waitlisting is still often seen. This bias is likely owing to short-term complications such as higher rates of wound complications, more extended hospital stays, and DGF [8] that could be associated with the BMI-based selection of transplant candidates endorsed by the practice guidelines of the American Society of Transplantation.

The correlation between having a high BMI and the postoperative outcomes of kidney transplantation has not been investigated yet in the Kingdom of Saudi Arabia. It is still little to no evidence that obese patients who have undergone kidney transplantation are devoid of any complications before, during, or after their procedure. Thus, this study’s aim is to shed light on the vagueness of the topic and hopefully help provide better conclusions for medical practitioners and the patients themselves.

## Materials and methods

This was a retrospective cross-sectional study, that was conducted by reviewing the charts of all the obese patients, whose BMI ≥ 30, who underwent kidney transplantation at King Abdullah Specialist Children's Hospital in Riyadh between January 2015 and March 2022. The total number of patients that were eligible and accessible for enrolment in the study was 142. Patients with a history of previous transplants, multi-organ transplant recipients with, a BMI less than 30, and those who underwent kidney transplants before 2015 were excluded. The reason 2015 was the cut-off is the unavailability of electronic records before 2015. The BESTCare system charts were reviewed by the co-authors after obtaining ethical approval from King Abdullah International Medical Research Centre (KAIMRC). The data were extracted on a google form, then recorded on an excel sheet designed to include all the variables needed. Outcome variables that were collected from the patient’s pre-operative assessment forms included demographic data, length of stay in the hospital, duration of dialysis, and presence of medical co-morbidities. Confidentiality of all patients was maintained throughout the study as no identifiers were collected, and each patient was assigned a serial number.

The data were collected, reviewed, and then fed to Statistical Package for Social Sciences version 21 (SPSS; IBM Corp., Armonk, NY). All statistical methods used were two-tailed with an alpha level of 0.05 considering significance if the p-value was less than 0.05. Descriptive analysis was done by prescribing frequency distribution and percentage for study variables including patients' body mass index, bio-demographic data, medical history, renal dialysis duration and causes, and renal transplant-related outcome (hospital stay, complications, graft function, and fate). Also, cross-tabulation for assessing the effect of patients' body mass index on their renal transplant surgery outcome was conducted using an exact probability test due to small frequency distributions. Serum creatinine level and BUN at different phases were displayed using the range, mean with standard deviation with repeated measures ANOVA for significance.

## Results

**Figure 1:**
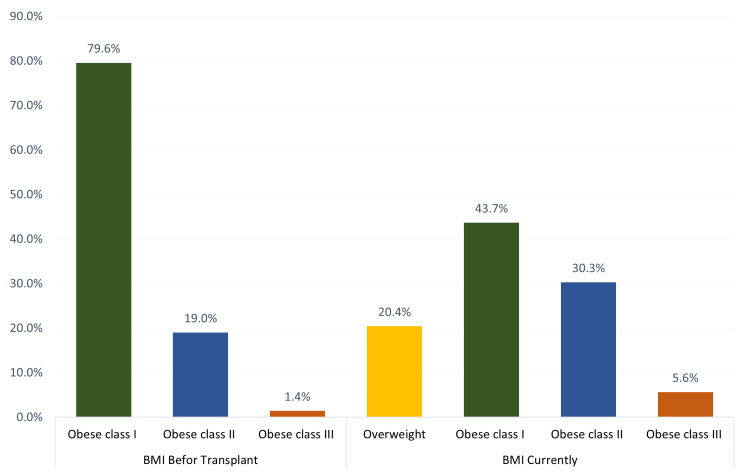
Patients’ body mass index before and after renal transplantation

A total of 142 patients fulfilling the inclusion criteria were included. Exact 141 were Saudi Arabian, and only one was non-Saudi. Regarding medical history, hypertension (HTN) was reported among 121 (85.2%), followed by dialysis (77.5%; 110), diabetes mellitus (DM) (52.1%; 74), dyslipidemia (24.6%; 35), endocrine diseases (15.5%; 22), and cardiovascular diseases, other than hypertension, (16.2%; 23). Exact 41 (39.4%) of the study patients had renal dialysis for less than two years, 41 (39.4%) for two to five years, and 22 (21.2%) for more than five years. As for the causes of ESRD, the most reported were DM (22.5%; 32), hypertension (10.6%;15), focal segmental glomerulosclerosis (FSGS) (6.3%; 9), and IgA nephropathy (4.9%; 7) (Table 1, Figure 1).

**Table 1:** Bio-demographic data of patients who have undergone kidney transplantation, King Abdullah Specialist Children's Hospital, Riyadh

Bio-demographic data		No	%
Nationality	Saudi	141	99.3
	Non-Saudi	1	0.7
Pre-surgical history	HTN	121	85.2
	Dialysis	110	77.5
	DM	74	52.1
	Dyslipidemia	35	24.6
	Endocrine Disease	22	15.5
	CVS	23	16.2
	Respiratory Disease	13	9.2
	Renal Disease	13	9.2
	Neurological Disease	10	7.0
	Hepatobiliary	9	6.3
	Rheumatological Disease	8	5.6
	MSK Disease	6	4.2
	Hematological Disease	3	2.1
	GIT Disease	3	2.1
	Oncological Disease	2	1.4
	Reproductive Disease	2	1.4
	Dermatology	1	0.7
	None	2	1.4
Duration of renal dialysis (years)	< 2 years	41	39.4
	2-5 years	41	39.4
	> 5 years	22	21.2
Cause of ESRD	Unknown	64	45.1
	DM	32	22.5
	HTN	15	10.6
	FSGS	9	6.3
	IgA nephropathy	7	4.9
	ADPKD	6	4.2
	CKD	2	1.4
	Nephrotic syndrome	2	1.4
	Atrophic kidney	2	1.4
	Lupus nephritis	1	0.7
	IgA Vasculitis	1	0.7
	Badet Bidel Syndrome	1	0.7
	Diffuse Global sclerosis with interstitial fibrosis and tubular atrophy	1	0.7
	Immune mediated Glomerulonephritis	1	0.7
	Posterior urethral valve	1	0.7
	Crescent forming glomerulonephritis (Ragnar)	1	0.7
	Reflux nephropathy	1	0.7
	Renal Agenesis	1	0.7
	Obesity	1	0.7

 

There was a significant difference between patients regarding pre-surgical history where all cases with class three obesity were hypertensive and on dialysis versus (77.8%; 21) and (70.4%; 19) of class two obesity and (86.7%; 98) and (78.8%; 89) of class one obesity cases, respectively (P = 0.041). There was no significant difference regarding ing duration of renal dialysis and causes of ESRD (Table 2).

**Table 2:** Bio-medical data by study patients’ pre-surgical body mass index P: Exact probability test, *P < 0.05 (significant)

Biomedical data		BMI Before Transplant						P-value
		Obese class 1		Obese class 2		Obese class 3		
		No	%	No	%	No	%	
Pre-surgical history	None	1	0.9	1	3.7	0	0.0	0.041*
	Cardiovascular Diseases	17	15.0	5	18.5	1	50.0	
	Respiratory Disease	11	9.7	2	7.4	0	0.0	
	Hematological Disease	3	2.7	0	0.0	0	0.0	
	Oncological Disease	2	1.8	0	0.0	0	0.0	
	Rheumatological Disease	3	2.7	5	18.5	0	0.0	
	Neurological Disease	5	4.4	5	18.5	0	0.0	
	Renal Disease	10	8.8	3	11.1	0	0.0	
	Reproductive Disease	2	1.8	0	0.0	0	0.0	
	Endocrine Disease	15	13.3	6	22.2	1	50.0	
	GIT Disease	2	1.8	1	3.7	0	0.0	
	MSK Disease	4	3.5	1	3.7	1	50.0	
	Hepatobiliary	6	5.3	2	7.4	1	50.0	
	Dermatology	0	0.0	1	3.7	0	0.0	
	Dialysis	89	78.8	19	70.4	2	100.0	
	Hypertension	98	86.7	21	77.8	2	100.0	
	Diabetes Mellitus type 2	59	52.2	14	51.9	1	50.0	
	Dyslipidemia	28	24.8	6	22.2	1	50.0	
Duration of renal dialysis (years)	< 2 years	35	41.2	4	23.5	2	100.0	0.194
	2-5 years	34	40.0	7	41.2	0	0.0	
	> 5 years	16	18.8	6	35.3	0	0.0	
Cause of ESRD	Unknown	54	47.8	10	37.0	0	0.0	0.704
	IgA nephropathy	5	4.4	1	3.7	1	50.0	
	HTN	14	12.4	1	3.7	0	0.0	
	DM	24	21.2	7	25.9	1	50.0	
	FSGS	6	5.3	3	11.1	0	0.0	
	Lupus nephritis	0	0.0	1	3.7	0	0.0	
	ADPKD	3	2.7	3	11.1	0	0.0	
	CKD	2	1.8	0	0.0	0	0.0	
	IgA Vasculitis	1	0.9	0	0.0	0	0.0	
	Badet Bidel Syndrome	1	0.9	0	0.0	0	0.0	
	Nephrotic syndrome	1	0.9	1	3.7	0	0.0	
	Diffuse Global sclerosis with interstitial fibrosis and tubular atrophy	1	0.9	0	0.0	0	0.0	
	Immune mediated Glomerulonephritis	1	0.9	0	0.0	0	0.0	
	Posterior urethral valve	1	0.9	0	0.0	0	0.0	
	Atrophic kidney	2	1.8	0	0.0	0	0.0	
	Crescent forming glomerulonephritis (Ragnar)	1	0.9	0	0.0	0	0.0	
	Reflux nephropathy	1	0.9	0	0.0	0	0.0	
	Renal Agenesis	0	0.0	1	3.7	0	0.0	
	Obesity	1	0.9	0	0.0	0	0.0	

A total of 93.7% (133) of the study patients had no pre-surgical complications which were 92% (104) for class one obesity, all of class two and three obesity patients (n = 27) with no statistical significance (P = 0.998) (Table 3).

**Table 3:** Pre-surgical complications by study patients' pre-surgical body mass index P: Exact probability test

Pre-surgical complications			BMI Before Transplant						P-value
	Total		Obese class 1		Obese class 2		Obese class 3		
	No	%	No	%	No	%	No	%	
None	133	93.7	104	92.0	27	100.0	2	100.0	0.998
Hypotension	1	0.7	1	0.9	0	0.0	0	0.0	
Pulmonary Edema	1	0.7	1	0.9	0	0.0	0	0.0	
Low Hb	1	0.7	1	0.9	0	0.0	0	0.0	
Gross hematuria	0	0.0	0	0.0	0	0.0	0	0.0	
Hypocalcemia	1	0.7	1	0.9	0	0.0	0	0.0	
Thrombosis right AV fistula	1	0.7	1	0.9	0	0.0	0	0.0	
Diabetic retinopathy, neuropathy, nephropathy	1	0.7	1	0.9	0	0.0	0	0.0	
Bilateral jugular vein occlusion	1	0.7	1	0.9	0	0.0	0	0.0	
Left ventricular clot	1	0.7	1	0.9	0	0.0	0	0.0	
Hyperkalemia	1	0.7	1	0.9	0	0.0	0	0.0	
Anuria	0	0.0	0	0.0	0	0.0	0	0.0	

As for the length of hospital stay, 35.9% (46) stayed for less than one week (36.3% of obese class one, 33.3% of obese class two, and 50% of obese class three; P = 0.789) while 25% (32) of the study cases stayed for more than two weeks (23.5% of obese class one, 33.3% of obese class two, and none of obese class three; P = 0.789). As for graft function, it was stable among 81.7% (116) of study cases (79.6% of obese class one, 88.9% of obese class two, and all obese class three; P = 0.692). Considering post-transplant complications, 14.1% (20) of the study cases had DM (16.8% of obese class one, 3.7% of obese class two, and none of obese class three; P = 0.996) and UTI among 7% (10) of the cases (6.2% of obese class one, 11.1% of obese class two, and none of obese class three; P = 0.996). As for patients' fate, only one case (0.7%) died which was obese class one. All these differences according to patients' BMI were statistically insignificant (Table 4).

**Table 4:** Post-transplant outcome among study patients by their body mass index P: Exact probability test

Outcome		Total		BMI Before Transplant						P-value
				Obese class 1		Obese class 2		Obese class 3		
		No	%	No	%	No	%	No	%	
Length of hospital stay	< 1 week	46	35.9	37	36.3	8	33.3	1	50.0	0.789
	1-2 weeks	50	39.1	41	40.2	8	33.3	1	50.0	
	> 2 weeks	32	25.0	24	23.5	8	33.3	0	0.0	
Graft Function	Stable	116	81.7	90	79.6	24	88.9	2	100.0	0.692
	Failure	2	1.4	2	1.8	0	0.0	0	0.0	
	Delayed graft function	2	1.4	1	0.9	1	3.7	0	0.0	
	Unknown	22	15.5	20	17.7	2	7.4	0	0.0	
Post-Transplant Complications	None	97	68.3	76	67.3	19	70.4	2	100.0	0.996
	HTN	3	2.1	2	1.8	1	3.7	0	0.0	
	DM	20	14.1	19	16.8	1	3.7	0	0.0	
	Dyslipidemia	3	2.1	1	0.9	2	7.4	0	0.0	
	Chronic low K	1	0.7	1	0.9	0	0.0	0	0.0	
	Loosing diarrhea	1	0.7	1	0.9	0	0.0	0	0.0	
	Hyperthyroidism	1	0.7	0	0.0	1	3.7	0	0.0	
	NASH	2	1.4	1	0.9	1	3.7	0	0.0	
	Acute tubular necrosis	3	2.1	2	1.8	1	3.7	0	0.0	
	Hypocalcemia	1	0.7	1	0.9	0	0.0	0	0.0	
	Hypercalcemia	1	0.7	1	0.9	0	0.0	0	0.0	
	Hypomagnesaemia / low mg	2	1.4	1	0.9	1	3.7	0	0.0	
	Membranous Nephropathy	1	0.7	1	0.9	0	0.0	0	0.0	
	Hydronephrosis	1	0.7	1	0.9	0	0.0	0	0.0	
	Kaposi’s Sarcoma	1	0.7	1	0.9	0	0.0	0	0.0	
	GU Complications	4	2.8	3	2.7	1	3.7	0	0.0	
	Neurological Complications	4	2.8	3	2.7	1	3.7	0	0.0	
	CVS Complications	4	2.8	2	1.8	2	7.4	0	0.0	
	Infectious Complications	3	2.1	3	2.7	0	0.0	0	0.0	
	MSK Complications	3	2.1	3	2.7	0	0.0	0	0.0	
	Dermatological Complications	2	1.4	2	1.8	0	0.0	0	0.0	
	Respiratory Complications	1	0.7	1	0.9	0	0.0	0	0.0	
	UTI	10	7.0	7	6.2	3	11.1	0	0.0	
Deceased / Alive	Alive	141	99.3	112	99.1	27	100.0	2	100.0	0.879
	Deceased	1	0.7	1	0.9	0	0.0	0	0.0	

Serum creatinine level showed a significant reduction from 845 ± 271.8 before transplant to 107.2 ± 97.0 one-year after surgery (P = 0.001). Also, BUN was significantly decreased from 21.6 ± 9.2 before surgery to 6.3 ± 3.7 one-year after transplant surgery (P = 0.001) (Table 5).

**Table 5:** Serum creatinine and BUN before, during and after renal transplant among study patients P: Repeated measures ANOVA, *P < 0.05 (significant)

Lab finding	Range	Mean	SD	P-value
Serum creatinine				0.001*
Before surgery	212-1,708	845.9	271.8	
At discharge	56-794	136.9	114.9	
6m post-surgery	56-707	110.5	82.0	
1-year post-surgery	53-883	107.2	97.0	
BUN				0.001*
Before surgery	4.8-44.7	21.6	9.2	
At discharge	2.8-38.7	9.5	6.4	
6m post-surgery	2.0-17.8	6.3	2.4	
1-year post-surgery	2.4-31.7	6.3	3.7	

## Discussion

When looking at the obesity classes before and after surgery, we found that the class one population had decreased in number by almost half after surgery. On the other hand, class three showed an increase in number. The decrease in class one was divided by either patients gaining or losing weight and then classified into their respective categories. One study supports the fact that successful transplantations are associated with higher weight and BMI without an elevation in lean body mass [9] These findings might help surgeons in looking at transplantation as an important element that influences weight. However, the reasons and factors determining whether a patient gains or loses weight are not clear from our data, due to its retrospective nature, and need further study. Furthermore, an important point to consider is waist circumference measurement since BMI alone or in combination with waist circumference could be associated with different results [10]. This supports the questionable role of BMI significance in determining the eligibility for transplantation since many centers reject patients based on BMI alone [11], and its lack of ability to differentiate between muscle mass and fat mass [12] puts its use into further question.

Our data shows that the most common condition in the population’s history was hypertension, yet the most common cause of ESRD was diabetes. This shows the major effect of diabetes before transplantation. After transplantation, however, cardiovascular disease has the largest impact on the survival of both the graft and the patient [13]. Lentine et al. found cardiovascular events increase with the increase in BMI [14]. The previous notion is important to keep in mind in that BMI could help predict associated comorbidities, and the fact that obesity is a risk factor for conditions such as hypertension [15], supports that idea. For classes one and two, we found a significant association between BMI and the patient being hypertensive or on dialysis, but a small fraction was free of them. In contrast, class three patients all had a history of being hypertensive. This finding could justify why some centers have a lower limit for transplants.

The majority of obese class one patient had no pre-renal transplant complications. However, some of the patient's experienced hypotension, anemia, hyperkalemia, and other pre-surgical complications. In the pre-operative assessment of obese patients who underwent bariatric surgery, it was found that obesity correlates with multiple comorbidities. These comorbidities can make intraoperative management difficult and complicate the post-operative course. Some obesity-related conditions include diabetes, gastroesophageal reflux disease, heart disease, and many others. Thus, it is important to evaluate such patients pre-operatively to identify these conditions and manage them accordingly to avoid post-operative complications [16]. On the other hand, all patients classified as obesity class two and class three did not present with pre-renal transplant complications.

Among our patients who were obese before undergoing renal transplant surgeries, obese class one patients have shown similarity in the length of hospital stay (LOS) by which 41 patients stayed for one to two weeks, followed by 37 patients that stayed for less than a week, and 24 patient stayed for more than two weeks. For obese class two patients, the length of stay was distributed equally between them in which eight patients stayed for less than a week, for one to two weeks, and more than two weeks. Only two patients with obesity class three stayed for less than a week and one to two weeks. This shows according to our data that there is no correlation between the progression of obesity class and the length of hospital stay which has been shown in another study that LOS is significantly longer in patients with BMI more than or equal to 35 compared to patients with BMI less than 35 [17]. As for graft function, most of the study cases had a stable function. Moreover, four cases of obesity class one and two have experienced either failure or DGF, and despite this study containing only two cases of obesity class three, all of them had stable graft function. It was documented in some articles that higher pre-transplant BMI was associated with the risk of DGF [18].

Post-transplant DM (PTDM) was the most notable complication post-transplant, and all PTDM cases were shown in obese class one patients except for one case which was in obese class two. PTDM is documented as a frequent post-transplant complication in allograft kidney recipients. It can be attributed to modifiable and non-modifiable risk factors. In non-modifiable risk factors, it is similar in the way of development of type two diabetes in the general population. However, modifiable risk factors can include perioperative stress, vitamin D deficiency, cytomegalovirus or hepatitis C, and immunosuppressive medications like glucocorticoids, mTOR inhibitors, and calcineurin inhibitors [19]. Urinary tract infections (UTI) were also noted post-transplant, they are an important factor that may lead to increased graft failure and morbidity. UTI occurs in 25% following one year of transplant in kidney transplant recipients and is responsible for 45% of infectious complications, which can worsen the quality of life and can potentially impair graft function [20]. Other post-transplant complications noted were hypertension, acute tubular necrosis, dyslipidemia, and others.

Both serum creatinine and blood urea nitrogen (BUN) have shown a significant reduction from the period preceding the surgery to the time of discharge in which the mean reduction was around sixfolds and twofolds for serum creatinine and BUN levels, respectively. A notable reduction was also noted after six months of discharge in both serum creatinine and BUN. Following one year after renal transplant surgery, there was nearly a plateau of mean values of both entities which were within the normal ranges.

## Conclusions

Our paper highlights the interrelation between successful transplantation and change in BMI classes post-op as an alteration in said classes was noted in the majority of our patients. However, no correlation has been established with regard to obesity class and length of hospital stay.

Hypertension was the most common comorbidity in our sample, yet diabetes was more likely to be the cause behind ESRD. PTDM was the most prominent post-transplant medical complication followed by UTI. With respect to kidney function, a remarkable reduction in serum creatinine and BUN has been observed at the time of discharge and after six months compared to pre-transplant measurements. DGF or failure was infrequent and has been noted in only a handful of cases, as most patients had stable grafts throughout their course.
